# Effectiveness of preoperative and perioperative pulmonary rehabilitation nursing program for the management of patients undergoing thoracic surgery: A systematic review and meta-analysis

**DOI:** 10.12669/pjms.40.6.9259

**Published:** 2024-07

**Authors:** Ming Xu, Xiaoqin Yang, Lingyan Guo

**Affiliations:** 1 Ming Xu, Department of Thoracic Surgery, Shanghai Pulmonary Hospital, 507 Zhengmin Road, Shanghai 200439, P.R. China; 2 Xiaoqin Yang, Department of Thoracic Surgery, Shanghai Pulmonary Hospital, 507 Zhengmin Road, Shanghai 200439, P.R. China; 3 Lingyan Guo, Department of Thoracic Surgery, Shanghai Pulmonary Hospital, 507 Zhengmin Road, Shanghai 200439, P.R. China

**Keywords:** Lung Cancer, Meta-Analysis, Pulmonary Rehabilitation

## Abstract

**Background & Objective:**

Several studies have investigated the effectiveness of preoperative or perioperative pulmonary rehabilitation in thoracic surgery patients, but the results are inconsistent and inconclusive. This study attempts to summarize the existing data on the effect of the preoperative and perioperative pulmonary rehabilitation nursing program for the management of patients undergoing thoracic surgery.

**Methods:**

Systematic search was done in PubMed Central, SCOPUS, EMBASE, MEDLINE, Google Scholar, and ScienceDirect for papers published until December 2022 and reporting data of postoperative complications and pulmonary health status in patients undergoing thoracic surgery and receiving preoperative or perioperative pulmonary rehabilitation nursing intervention or standard care. Meta-analysis was done by random-effects model and pooled standardised mean differences (SMD) or odds ratios (OR) along with 95% confidence intervals (CIs) were reported.

**Results:**

Eighteen studies were included and analysed. Pooled SMD was 0.44 (95%CI: -0.21 to 1.08) for forced expiratory volume (FEV-1), -0.34 (95%CI: -0.94 to 0.26) for peak expiratory flow (PEF), 0.61 (95%CI: -0.60 to 1.81) for forced vital capacity (FVC), 0.42 (95%CI: -0.13 to 0.98) for diffusing capacity of carbon monoxide (DLCO). Pooled SMD for length of hospital stay was -0.64 (95%CI: -1.09 to -0.19). Pooled OR was 0.87 [95%CI: 0.32 to 2.37] for all-cause mortality, 0.35 [95%CI: 0.25 to 0.50] for postoperative pulmonary complications, 0.98 [95%CI: 0.45 to 2.12] for respiratory failure, 0.52 [95%CI: 0.38 to 0.78] for pneumonia and 0.50 [95%CI: 0.33 to 0.76] for atelectasis.

**Conclusion:**

Perioperative pulmonary rehabilitation nursing program is effective in reducing the postoperative lung complications and shortening the length of hospital stay in patients undergoing thoracic surgery.

## INTRODUCTION

Thoracic surgery, such as lung resection and esophagectomy, is a common surgical procedure performed to treat various thoracic conditions. However, this surgery has a high risk of postoperative complications, particularly respiratory ones. Studies have reported that the incidence of postoperative respiratory complications after thoracic surgery ranges from 20-50%.^1^,^2^ These complications can prolong hospital stay, increase the risk of readmission and lead to long-term morbidity.^3^,^4^ Some of the common respiratory complications following thoracic surgery include atelectasis, pneumonia, and respiratory failure.^5^,^6^ Therefore, proper management of patients before and after the thoracic surgery is crucial to minimize the risk of postoperative complications.

From the time the patient decides to have surgery, pulmonary rehabilitation nurses start providing pre-rehabilitation for two to four weeks or less prior to hospital admission, followed by post-surgery rehabilitation and continued rehabilitation after the discharge. The main components of rehabilitation include smoking cessation, correction of anemia, aerobic exercises, resistance strength training, inspiratory muscle training (IMT), optimization of nutrition and psychological support.^7^

Preoperative and perioperative pulmonary rehabilitation have been shown to be useful in preventing postoperative complications in patients undergoing thoracic surgery. Pulmonary rehabilitation, an evidence-based program of exercise and education, can enhance the lung function, ability to exercise and improve the life quality of chronic lung disease patients. A pulmonary rehabilitation medical team that adapts a multidisciplinary model of thoracic medical care, nutrition and rehabilitation will be able to provide a personalised health education model for perioperative patients and thus, reduce the risk of postoperative respiratory complications, such as atelectasis, pneumonia, and respiratory failure.^8^,^9^Occordion, R. Satava, et al., A. Kazaryan, et al. Additionally, preoperative or perioperative pulmonary rehabilitation has been shown to shorten the duration of hospitalization and improve postoperative functional outcomes.^10^

Several studies have demonstrated the effectiveness of preoperative or perioperative pulmonary rehabilitation in thoracic surgery patients. A randomized controlled trial (RCT) by He et al 2018^11^ showed that preoperative pulmonary rehabilitation has lowered the rate of postoperative complications and the duration of hospitalization in patients undergoing lung resection. Another randomized controlled trial conducted by Lai Y et al. 2016^12^ showed that a perioperative pulmonary rehabilitation program improved lung function and exercise capacity and reduced the incidence of postoperative complications. These studies demonstrate the potential benefits of preoperative or perioperative pulmonary rehabilitation in patients undergoing thoracic surgery.

While several studies have investigated the effectiveness of preoperative or perioperative pulmonary rehabilitation in thoracic surgery patients, their results are inconsistent and inconclusive. The current systematic review and meta-analysis aimed to evaluate the efficacy of preoperative or perioperative pulmonary rehabilitation in preventing postoperative complications of thoracic surgery, and to provide a clear and reliable guideline to the healthcare providers.

## METHODS

We conducted a systematic literature search in PubMed Central, SCOPUS, EMBASE, MEDLINE, Google Scholar, and ScienceDirect databases. Both medical subject headings (MeSH) and free-text headings were combined to form the search strategy. Appropriate Boolean operators (“AND” & “OR” & “NOT”) were used between the predefined search terms. Studies from January 1964 to December 2022 without any language restrictions were considered.

### Inclusion criteria:

### Study design:

The study included RCTs, non-randomized controlled trials, and observational studies. It is registered in PROSPERO with number CRD42023395349.

### Study participants:

Studies done in the patients undergoing thoracic surgery of any type and comorbidity status were included.

### Intervention and comparison:

Studies comparing the effect of preoperative or perioperative pulmonary rehabilitation nursing intervention for preventing postoperative complications and improve the pulmonary health status of the patients as opposed to the usual or standard care were included.

### Outcomes:

Forced expiratory volume [FEV1], peak expiratory flow [PEF], forced vital capacity [FVC], diffusing capacity of carbon monoxide [DLCO], mortality, pulmonary complications (respiratory failure, pneumonia, atelectasis), length of hospitalization and ICU stay, and the quality of life.

### Study selection:

During the initial stage of study selection, title, keywords, and abstracts of the studies were independently screened by two investigators. At the second stage, both investigators reviewed the full texts of the retrieved and shortlisted studies and selected those that met the eligibility criteria for the analysis. The review followed 2020 “Preferred Reporting Items for Systematic Reviews and Meta-Analyses (PRISMA) checklist”.^13^

### Extracting data:

Once the list of eligible full-text articles for the review was finalized, the two investigators conducted a manual data extraction process using a pre-defined semi-structured data collection form. The first author recorded the data, and the second author reviewed the data entry for accuracy.

### Risk of bias:

Study quality was assessed by two investigators using the Cochrane risk of bias tool (RoB2) for RCTs and the Newcastle Ottawa (NO) scale for observational studies.^14^,^15^ Based on the assessment criteria, studies were categorized as having low, high, or somewhat concerning risk of bias.

### Statistical analysis:

The statistical analysis for this review was conducted using STATA version 14.2. The continuous outcomes were analysed for each group by obtaining the mean, standard deviation (SD), and total sample size. The standardized mean difference (SMD) with 95% confidence interval (CI) measured the pooled effect. For binary outcomes, the odds ratio (OR) with 95%CI was calculated. The random effects model with inverse variance method was used.^16^ Chi-square test with I2 statistic were used to assess heterogeneity. Sensitivity analysis was conducted to identify the effects of single studies on the pooled estimates. Publication bias assessment and meta-regression were performed for outcomes that had at least 10 studies. The funnel plot and Egger’s test^16^ assessed publication bias.

## RESULTS

### Search results

**Fig.1:**
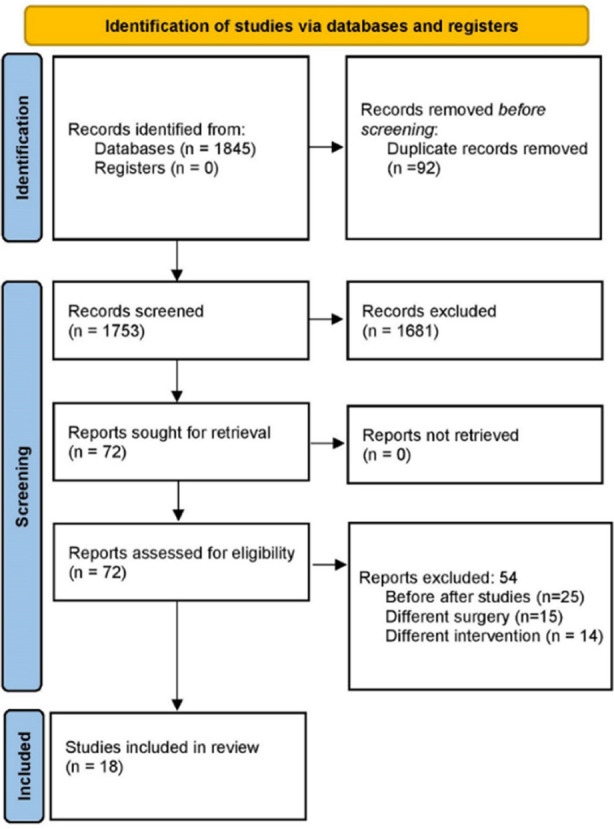
Search strategy.

In total, 1845 citations from various databases were identified by the initial screening. After removing duplicates, 78 full-text studies remained, which were further reduced to 72. Additional four articles were identified by searching the bibliography of the screened studies. After the secondary screening process, we analysed data from 18 studies that met the eligibility criteria (Fig.1).^11^,^12^,^17^–^32^

### Characteristics of the included studies:

Half of the selected studies were conducted in Asian countries like China (seven studies) and Japan (two studies). Most (10 studies) were RCTs, while the rest were either prospective or retrospective cohort studies. Half of the studies had participants with lung cancer and COPD. Sample sizes in the intervention arm varied between 9-197, while sample size in control arm varied between 8-742, (Table-I). Most studies (7/10 RCTs) had higher risk of bias, while most observational studies (6/8) were of good to satisfactory quality as per NO scale for observational studies.

**Table-I:** Characteristics of the included studies

First author and year	Study design	Country	Intervention details	Type of surgery	Study participants	Sample size	Risk of bias
Benzo et al 2011	RCT	USA	Preoperative PR consisting of lower and upper extremity endurance training, strengthening exercises	Lung cancer resection	Lung cancer patients with moderate to severe COPD	I=9 C=8	High risk
Boujibar et al 2018	Cohort study	France	Rehabilitation program consisted of exercise retraining, muscular strengthening of lower and upper limbs, therapeutic education and help with smoking cessation	Pulmonary lobectomy	NSCLC	I=19 C=15	Low risk
Bradley et al 2013	Cohort study	UK	PR program consisting of exercise classes, smoking cessation, dietary advice and patient education	Lung cancer surgery	Lung cancer patients	I=58 C=305	Low risk
Chesterfield-Thomas et al 2016	Prospective	UK	PR program consisting of respiratory muscle training, breathing exercises, Cardiovascular exercises, education and pharmacology agents	Pulmonary resection	Lung cancer patients	I=33 C=9	Low risk
Glogowska et al 2017	Prospective	Poland	Innovative algorithm of perioperative intensive physiotherapy until discharge from hospital	Thoracic surgery	Lung cancer patients	I=68 C=51	Low risk
He et al 2018	RCT	China	Preoperative PR program	Open thoracotomy	NSCLC with COPD patients	I=55 C=55	High risk
Lai et al 2016	RCT	China	PR program consisting of pharmacotherapy with physical endurance training and respiratory training	Pneumonectomy	Lung cancer patients with COPD	I=26 C=29	Some concerns
Lai Y et al 2016	RCT	China	Systematic and intensive preoperative PR program focusing on exercise endurance training and inspiratory muscle training	Lobectomy	NSCLC patients	I=30 C=30	Some concerns
Lai et al 2017	RCT	China	PR program consisting of thoracic expansion and incentive spirometry exercises, abdominal breathing, aerobic endurance exercises	Lobectomy	Elderly lung cancer patients	I=51 C=50	High risk
Licker et al 2016	RCT	Switzerland	Preoperative PR program based on high- intensity interval training (HIIT)	Lung resection	NSCLC patients	I=74 C=77	High risk
Meng et al 2018	NR	China	Preoperative short term high intensity lung rehabilitation program	Lobectomy	Lung cancer patients with COPD	I=43 C=58	Low risk
Mujovic et al 2015	Prospective	Serbia	Preoperative PR program based on physiotherapy exercises	Lung resection	NSCLC with COPD	I=56 C=47	Low risk
Pehlivan et al 2011	RCT	Turkey	Intensive physical therapy consisting of chest physiotherapy and walking exercise	Lung cancer resection	Lung cancer patients	I=30 C=30	High risk
Saito et al 2017	Retrospective	Japan	Breathing and coughing techniques, instructed on incentive respiratory exercise, and practiced peripheral muscle exercise training	Lung resection	NSCLC with COPD	I=31 C=31	High risk
Sekine et al 2005	RCT	Japan	Perioperative rehabilitation and physiotherapy	Lobectomy	NSCLC with COPD	I=22 C=60	Some concerns
Stefanelli et al 2013	RCT	Italy	Respiratory exercises on the bench, mattress pad and wall bars followed by high intensity training of upper limbs and lower limbs	Lobectomy	NSCLC with COPD	I=20 C=20	High risk
Zhang et al 2014	RCT	China	Preoperative rehabilitation exercise training	Open thoracotomy	NSCLC with COPD	I=43 C=43	High risk
Zhou et al 2017	Cohort study	China	PR program consisting of Inspiratory muscle training, education, aerobic endurance training	Lobectomy	Lung cancer patients	I=197 C=742	High risk

I – Intervention; C – Control; NR – Not reported; RCT – Randomized controlled trial; NSCLC – Non-Small Cell Lung Cancer; COPD – Chronic Obstructive Pulmonary Disease; UK – United Kingdom; USA – United States of America; PR – Pulmonary Rehabilitation.

### Pulmonary function tests:

### FEV1:

Six studies provided information on the difference in FEV1 between the groups. The pooled SMD was 0.44 (95%CI: -0.21 to 1.08; I2=89.4%), meaning no difference between the groups (p=0.18).

### PEF:

A total of three studies showed the differences in PEF between the pulmonary rehabilitation and the control group. The pooled SMD was -0.34 (95%CI: -0.94 to 0.26; I^2^=79.1%; p=0.27).

### FVC:

Two studies reported differences in FVC between both groups of patients. The pooled SMD was 0.61 (95%CI: -0.60 to 1.81; I^2^=90.3%), which meant that this parameter was similar in two groups (p=0.32).

### DLCO:

Two studies provided information on the difference in DLCO between the rehabilitation and the control group. The pooled SMD was 0.42 (95%CI: -0.13 to 0.98; I^2^=67.4%) (p=0.14).

### All-cause mortality:

**Fig.2:**
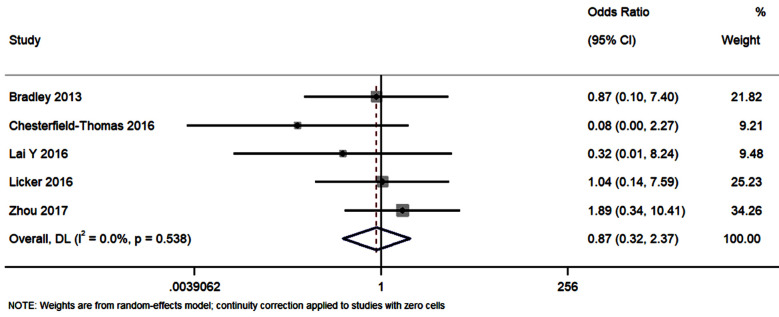
Forest plot showing the effectiveness of preoperative and perioperative pulmonary rehabilitation nursing program on all-cause mortality.

Five studies had data on the disparity in all-cause mortality between the pulmonary rehabilitation and the control group of patients. The pooled OR was 0.87 [95%CI: 0.32 to 2.37; I^2^=0%]. This shows that patients receiving pulmonary rehabilitation nursing intervention did not have significant variation in all-cause mortality between the groups (p=0.79) (Fig.2).

### Postoperative Pulmonary complication (overall):

**Fig.3:**
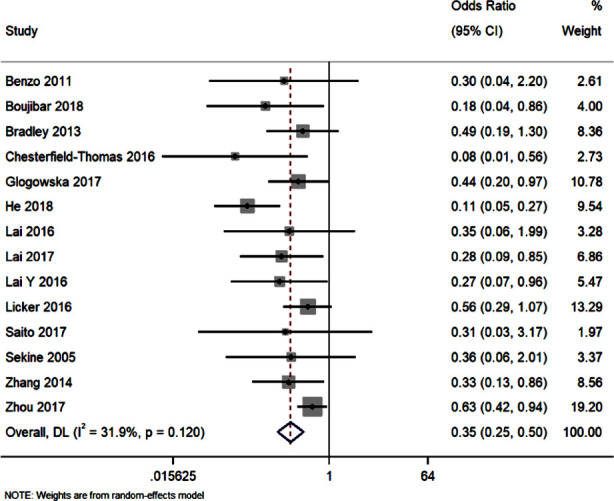
Forest plot showing the effectiveness of preoperative and perioperative pulmonary rehabilitation nursing program on postoperative pulmonary complications.

Fourteen studies reported data on the rate of postoperative pulmonary complication in patients who received preoperative or perioperative pulmonary rehabilitation nursing intervention and the control group. The pooled OR was 0.35 [95%CI: 0.25 to 0.50; I^2^=31.5%]. These results show that patients receiving pulmonary rehabilitation nursing intervention had significant lower rate of postoperative pulmonary complications (p<0.001) (Fig.3). Publication bias assessment showed that the funnel plot was asymmetrical with statistically significant Egger’s test (p=0.01)

### Postoperative Respiratory failure:

Eight studies have reported on the disparity in postoperative respiratory failure in two groups. The pooled OR was 0.98 [95%CI: 0.45 to 2.12; I^2^=0%]. This shows that patients receiving pulmonary rehabilitation nursing intervention and the control group had comparable rate of postoperative respiratory failure (p=0.96).

### Pneumonia:

**Fig.4:**
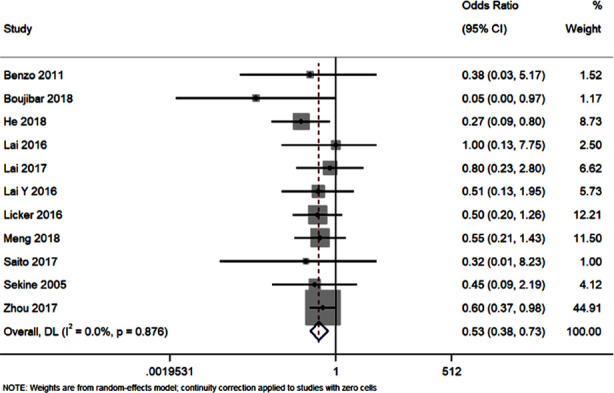
Forest plot showing the effectiveness of preoperative and perioperative pulmonary rehabilitation nursing program on pneumonia.

Eleven studies have reported on the differences in postoperative pneumonia in patients receiving different modes of nursing. The pooled OR was 0.53 [95%CI: 0.38 to 0.73; I^2^=0%]. Patients receiving pulmonary rehabilitation nursing intervention had significant reduction in postoperative pneumonia rate (p<0.001) (Fig.4). Funnel plot was slightly asymmetrical. However, Egger’s test was non-significant (p=0.17)

### Atelectasis:

Nine studies have reported on the disparity in postoperative atelectasis between the two groups. The pooled OR was 0.50 [95%CI: 0.33 to 0.76; I^2^=0%]. These results indicate that compared to routine nursing, pulmonary rehabilitation nursing intervention is associated with a significant reduction in postoperative atelectasis rate (p=0.001).

### Length of hospital stay:

**Fig.5:**
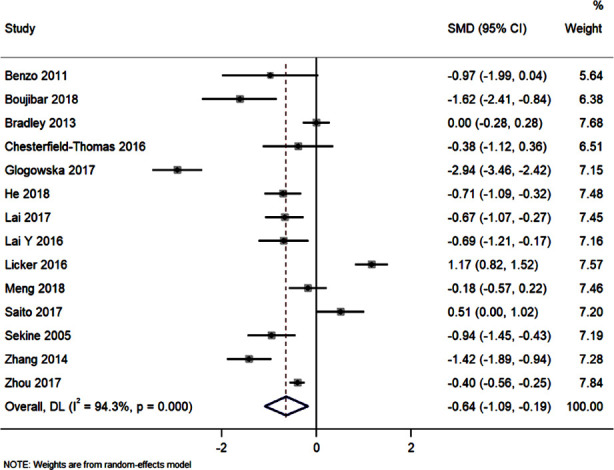
Forest plot showing the effectiveness of preoperative and perioperative pulmonary rehabilitation nursing program on length of hospital stay.

Length of hospital stay in pulmonary rehabilitation and the control groups of patients was evaluated in 14 studies. Compared to control, pulmonary rehabilitation was associated with a statistically significant reduction in length of hospital stay (pooled SMD -0.64 (95%CI: -1.09 to -0.19; I^2^=94.3%; p=0.006) (Fig.5). Funnel plot was slightly asymmetrical. However, Egger’s test was non-significant (p=0.32). Meta-regression was performed using the covariates such as study design, participants, study region, and quality of the studies to explore the high heterogeneity. However, none of these factors were found to be a source of heterogeneity.

### Length of ICU stay:

Three studies reported the disparity in the length of ICU stay between the groups. The pooled SMD was -0.45 (95%CI: -1.17 to 0.27; I^2^=88.3%; p=0.22).

### Quality of life:

Two studies reported the difference in quality of life. The pooled SMD was 0.15 (95%CI: -0.16 to 0.46; I^2^=0%) signifying no intergroup difference (p=0.35).

## DISCUSSION

The current study aimed to summarize and evaluate existing literature to investigate the effectiveness of a preoperative and perioperative pulmonary rehabilitation nursing program for the management of patients undergoing thoracic surgery.

In total, 18 studies were included. Most eligible studies were RCTs and had higher risk of bias, while most included observational studies had lower risk of bias. Our results suggest that the intervention is effective in reducing postoperative pulmonary complications especially pneumonia, atelectasis, and respiratory failure. We also showed that the intervention can markedly lower the duration of hospitalization.

However, the intervention did not result in significant differences in terms of pulmonary function tests like FEV1, PEF and FVC. The findings of this study are in agreement with the previous reviews that have also demonstrated a significant impact of pulmonary rehabilitation nursing intervention on reducing postoperative pulmonary complication.^33^,^34^

We may speculate that the efficiency of intervention in reducing postoperative pulmonary complications and the length of hospital stay is due to its focus on improving patients’ lung function and overall fitness before and after the surgery. The preoperative component of the program can help to identify and address any underlying respiratory issues, such as chronic obstructive pulmonary disease (COPD), that may have led to higher risk of complications.^33^ The perioperative component of the program likely helps to maintain and improve lung function during the recovery period, which can be critical for preventing complications such as pneumonia, atelectasis and respiratory failure, and subsequently, result in shorter hospital stay. Additionally, the program can be very useful in educating patients and their families on how to properly manage their breathing and cough after the surgery, which can further help to prevent complications and shorten the length of hospitalization. Finally, implementing the program may improve overall physical fitness of the patient, which can help to reduce the risk of complications after the surgery^34^ and shorten the time to discharge.^35^

Nurses played a primary role in providing rehabilitation intervention across almost all the included studies. Nurses were either in a full-time role exclusively for the provision of pulmonary rehabilitation intervention or provided rehabilitation in addition to their other responsibilities.

Our study showed no significant intergroup difference in pulmonary function tests, which may suggest that the intervention did not have a direct impact on lung function. Pulmonary rehabilitation programs are designed to improve lung function, but their effect on pulmonary function test parameters may vary with the patient’s baseline lung function, the type of surgery, and other factors. Further studies are needed to determine the precise mechanisms by which the intervention improves lung function and whether it leads to significant changes in pulmonary function test parameters.

### Strength of the study:

The main strength of this review is the meticulous search strategy which was free from any language restrictions, thus making the review more comprehensive and trustworthy. Furthermore, our search included studies printed up to the year 2022 to provide the best possible evidence.

### Limitations

Our meta-analysis has certain limitations that need to be considered while interpreting the results. Methodological and quality differences among the included studies could have influenced the results. Additionally, we detected a significant difference between-study variability for some outcomes. Meta-regression analysis failed to identify the source of heterogeneity. Publication bias assessment revealed an asymmetrical funnel plot for some outcomes, which might have an impact on the credibility of the findings.

## CONCLUSIONS

Despite some limitations, our study has some important implications for oncologists, pulmonologists, and nursing care professionals. We demonstrate clear benefits of implementing preoperative or perioperative pulmonary rehabilitation programs to reduce the postoperative pulmonary complications, thereby leading to a decrease in the length of hospital stay. The healthcare facilities should focus on designing pulmonary rehabilitation interventions that are aimed at providing support to the patients, thereby reducing the morbidity and mortality associated with pulmonary complications.

### Authors’ contributions:

**MX and XY** conceived and designed the study.

**MX, XY and LG** collected the data and performed the analysis.

**MX and XY** were involved in the writing of the manuscript and is responsible for the integrity of the study.

All authors have read and approved the final manuscript.
